# miR-612 negatively regulates colorectal cancer growth and metastasis by targeting AKT2

**DOI:** 10.1038/cddis.2015.184

**Published:** 2015-07-09

**Authors:** L Sheng, P He, X Yang, M Zhou, Q Feng

**Affiliations:** 1 Department of Emergency Medicine, Shanghai Jiao Tong University Affiliated Sixth People's Hospital, Shanghai, China; 2 Department of Gastroenterology, Shanghai Jiao Tong University Affiliated Sixth People's Hospital, Shanghai, China

## Abstract

Colorectal cancer (CRC) is one of the most common cancers worldwide, with a particularly high incidence in developed countries. Distant metastasis and recurrence are the main causes of CRC-related deaths. MicroRNAs (miRNAs) in the serum make them potential biomarkers for cancers, as reported in serum or tumor tissues from CRC patients. In this study, we found that miR-612 expression was significantly lower in CRC tissues or cells compared with peritumor tissues or normal cells, and lower in metastatic CRC specimens compared with non-metastatic specimens, whereas AKT2 exhibited opposite trend. Gain-of-function and loss-of-function assays showed that miR-612 inhibited CRC cell proliferation and migration *in vitro* by Cell Counting Kit-8 and transwell assays. Further analysis revealed that miR-612 directly suppressed AKT2, which in turn inhibited the downstream epithelial–mesenchymal transition-related signaling pathway. These results were additionally validated *in vivo* by tumorigenesis and liver metastasis experiments. The results of this study suggested a critical role of miR-612 in the development of CRC.

Colorectal cancer (CRC) is the third most common cancer in both males and females worldwide, accounting for about 10% of all cancer cases, with over a million new cases diagnosed worldwide annually.^1, 2^ CRC is more common in developed countries, where more than 65% of total cases occur, primarily because of lifestyle factors. CRC is the second most frequent cause of cancer-related deaths in the United States and Europe. The major cause of death from CRC is metastatic disease.^3^ The 5-year survival rate is ~90% in early CRC patients, but decreases to <5% in patients with distant metastases.^4, 5^ CRC development involves a multistep process with the accumulation of both genetic and non-genetic risk factors, including older age, male sex, high intake of fat, alcohol or red meat, obesity, smoking, and a lack of physical exercise.^6, 7, 8^ In addition, recent studies focus on the mechanism of CRC metastasis, finding out that several pathways including the phosphoinositide 3-kinase (PI3K)/AKT signaling pathway and Wnt signaling pathway have essential roles in cell survival and metastasis in CRC.^9, 10^ However, the molecular mechanism responsible for CRC occurrence remains obscure.

MicroRNAs (miRNAs) constitute a class of small endogenous noncoding RNAs of 22 or so nucleotides, which negatively regulate gene expression by binding to the 3′-untranslated region (UTR) of their target mRNAs. Perfect complementarity between the miRNA and the 3′-UTR of the target transcript leads to degradation of the mRNA, whereas partial complementarity results in inhibition of translation. The majority of interactions between miRNAs and mRNAs in animals are only partially complementary, and translational inhibition is therefore the most common result.^11^ The involvements of miRNAs in carcinogenesis and tumor progression have been confirmed by numerous functional studies.^12^ Accumulated evidence has emphasized the pervasive effects of miRNAs on CRC tumorigenesis, including oncogenesis, angiogenesis, progression, invasion, and metastasis.^13, 14^ A series of miRNAs was found to be aberrantly expressed in CRC compared with normal colon mucosa, with the upregulation of miR-18a, miR-31, miR-155, miR-223, and miR-224, and downregulation of miR-192, miR-215, miR-345, miR-601, and miR-612.^15, 16, 17^ Many of these miRNAs have confirmed functions in CRC development and some have been used as markers for predicting treatment response. For instance, upregulated miR-10b and miR-192/215 were reported to indicate chemosensitivity to 5-fluorouracil-based chemotherapy.^18, 19^ Additionally, miR-18a downregulation was associated with poor survival in CRC patients, and its expression predicted progression-free survival in epidermal growth factor receptor-targeted therapy.^20^ Overall, these results indicate a link between miRNAs and the development of CRC.

Previous research suggested a role for miR-612 in tumorigenesis. miR-612 levels in hepatocellular carcinoma (HCC) patients were inversely associated with tumor size, stage, epithelial–mesenchymal transition (EMT), and metastasis.^21^ Importantly, miR-612 inhibited cell proliferation, migration, invasion, and metastasis by targeting AKT2 in HCC, suggesting its negative role in tumor development.^21^ However, the role of miR-612 in CRC pathogenesis remains unknown. In the current study, we found that miR-612 expression was downregulated in CRC specimens and CRC cell lines, and was low as well in metastatic CRC samples compared with non-metastatic samples. Functional assays showed that miR-612 inhibited the expression of proteins involved in EMT progression, thereby suppressing CRC cell growth, as a consequence of impaired expression of AKT2. *In vivo* assay validated that miR-612 inhibited CRC occurrence and liver metastasis. Our results demonstrated the critical role of miR-612 in CRC growth and offered new insights into the regulation of CRC.

## Results

### miR-612 expression was downregulated in CRC samples and CRC cells

**Figure 1:**
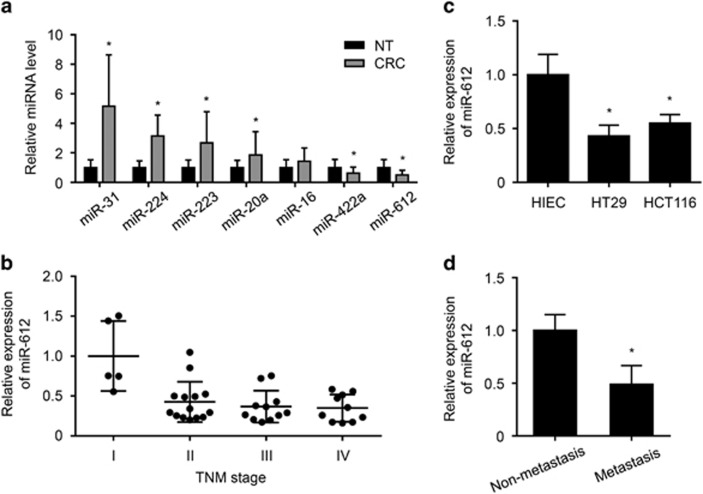
Expression of miR-612 was negatively correlated with CRC occurrence and metastasis. (**a**) Real-time PCR was used to detect the relative expression of several miRNAs in the CRC tissue specimens compared with adjacent NT specimens. Data were normalized to the expression level in NT specimens (*n*=40 per group). (**b**) Real-time PCR was used to detect the relative expression of miR-612 in the CRC tissue specimens of different tumor lymph node metastasis (TNM) stage. Data were normalized to the expression level of the mean value of group I (*n*≥5 per group). (**c**) Real-time PCR was used to measure relative expression of miR-612 CRC cell lines, HT29 and HCT116, compared with the normal HIECs. Data were normalized to the expression level in HIECs (*n*=3). (**d**) Real-time PCR was used to measure relative expression of miR-612 in non-metastatic and metastatic CRC specimens. Data were normalized to the expression level in non-metastatic tissues (*n*=20 per group). All the data were presented as mean±S.D. from three independent experiments. *P<0.05

We investigated the role of miR-612 in CRC occurrence by detecting its expression, together with that of several other miRNAs, in 40 CRC specimens and 40 normal tissue (NT) specimens adjacent to tumors. The clinicopathologic characteristics of CRC patients were shown in Table 1. Consistent with previous studies, miR-31, miR-224, miR-223, and miR-20a were upregulated in CRC samples compared with NT samples, whereas miR-612 expression was significantly decreased (Figure 1a). Moreover, we found that miR-612 expression was correlated significantly with CRC severity (Spearman's *R*s=−0.392, *P*=0.012) (Figure 1b). Consistently, miR-612 expression was lower in HT29 and HCT116 CRC cells compared with normal human intestinal epithelial cells (HIECs) (Figure 1c). In addition, miR-612 was downregulated in CRC tissues from metastatic cases compared with non-metastatic cases (Figure 1d) and correlated significantly with tumor metastasis (Spearman's *R*s=−0.418, *P*=0.007). These results indicated that miR-612 expression was negatively correlated with occurrence and metastasis of CRC.

### miR-612 modulated proliferation and migration activity of CRC cells by regulating EMT progression

**Figure 2:**
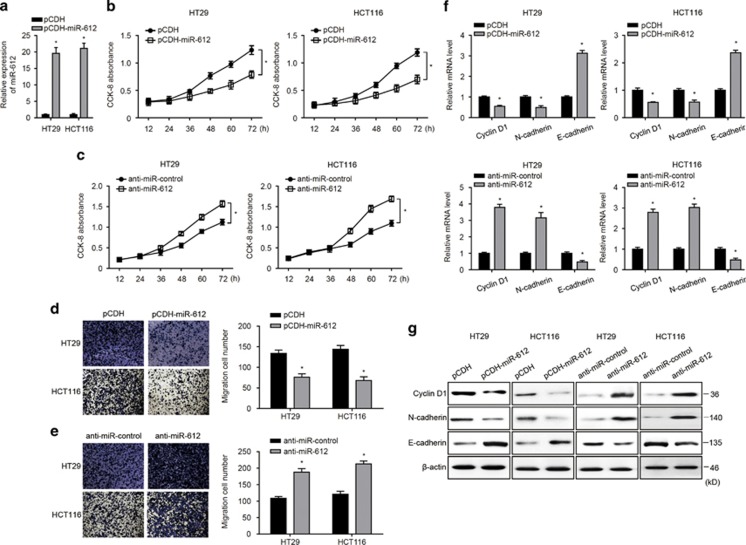
miR-612 regulated proliferation, migration, and EMT progression of CRC cells. (**a**) miR-612 was overexpressed in two CRC cell lines, HT29 and HCT116. Real-time PCR was conducted to detect the expression of miR-612. (**b**) Proliferation activity was conducted by CCK-8 assay at the indicated time after seeding (*n*=3). (**c**) Anti-miR-612 was used to inhibit miR-612 in CRC cells. Proliferation activity was conducted by CCK-8 assay at the indicated time after seeding (*n*=3). (**d**) miR-612 was overexpressed in two CRC cell lines. The indicated cells were seeded into the upper uncoated inserts with serum-free medium. Cells were allowed to migrate for 12 h at 37 °C. The migrated cells were observed using a microscope and counted. Representative images are shown on the left and summarized results are shown as mean±S.D. from three independent experiments (*n*=3) on the right. (**e**) Cells transfected with anti-miR-612 were plated at day 2 after transfection. Medium containing 10% FBS was added to the lower chamber. Cells were allowed to migrate for 12 h at 37 °C. The migrated cells were observed using a microscope and counted. Representative images are shown on the left and summarized results are shown as mean±S.D. from three independent experiments (*n*=3) on the right. (**f**–**g**) The expression of cyclin D1, E-cadherin, and N-cadherin was detected by real-time PCR (**f**) and western blotting (**g**) in the indicated CRC cells (*n*=3). All the data were presented as mean±S.D. from three independent experiments. *P<0.05

Given that miR-612 was aberrantly expressed in CRC specimens, we hypothesized that miR-612 may regulate CRC growth and metastasis. To test our hypothesis, we overexpressed miR-612 by lentivirus-containing miR-612-specific sequence or inhibited it using an miR-612-specific antisense oligonucleotide inhibitor (anti-miR-612). After determining the alteration of miR-612 expression (Figure 2a), we performed Cell Counting Kit-8 (CCK-8) assay at 12, 24, 36, 48, 60, and 72 h postseeding. Thus, we found out that the ectopic miR-612 expression significantly inhibited the proliferation activity in both CRC cells (Figure 2b), whereas miR-612 inhibition augmented CRC cell growth (Figure 2c). Additionally, the transwell assay was conducted to evaluate the cell migration activity, with the results that forced expression of miR-612 inhibited CRC cell migration (Figure 2d), whereas suppression of miR-612 facilitated cell migration (Figure 2e). These results suggest that miR-612 is involved in the proliferation and migration activity of CRC cells. We therefore evaluated several putative EMT-related and proliferation markers by real-time PCR and western blotting. Both mRNA and protein levels of N-cadherin and cyclin D1 were significantly reduced by miR-612 overexpression and increased by miR-612 inhibition, whereas E-cadherin, which was supposed to be downregulated during EMT progression, was upregulated in miR-612-overexpressing cells and downregulated in miR-612-inhibited cells (Figures 2f and g). These data indicate that miR-612 regulated CRC cell proliferation and migration by modulating the expression of EMT- and proliferation-related proteins.

### AKT2 was a target of miR-612 in CRC cells

**Figure 3:**
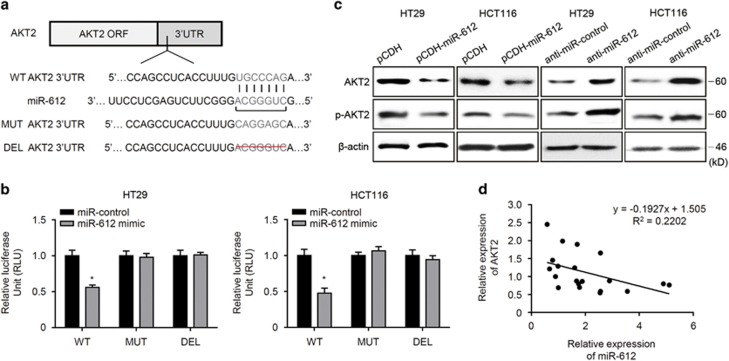
miR-612 directly regulated AKT2 expression in CRC cells. (**a**) The wt, mutated, or deleted AKT2 3′-UTR was transfected into CRC cells with or without synthetic miR-612 mimic. (**b**) Luciferase activity was determined 36 h after transfection. Data were normalized to the luciferase activity transfected with miR-control (*n*=3). (**c**) Expression of AKT2 and p-AKT2 upon miR-612 overexpression or miR-612 inhibition was detected in two CRC cell lines by western blotting. (**d**) Relationship of relative miR-612 and AKT2 expression. We have drawn the correlation formula: *y*=−0.1927*x*+1.505 (*x* is the expression level of miR-612, *y* is the expression level of AKT2) (*n*=20). All the data were presented as mean±S.D. from three independent experiments. *P<0.05

AKT2 is known to promote EMT progression, thereby inhibiting cancer growth and metastasis. We searched publicly available miRNA-target prediction websites and found that miR-612 could target AKT2. To confirm if AKT2 was a direct target of miR-612 in CRC cells, we conducted luciferase reporter assays after transfection of wild type (wt), mutated, or deleted AKT2 3′-UTR into CRC cells, with or without synthetic miR-612 mimic (Figure 3a). miR-612 reduced luciferase activity in cells transfected with wt AKT2 3′-UTR, but had no effect on luciferase activity in cells transfected with mutant or deleted AKT2 3′-UTR (Figure 3b). Additionally, miR-612 regulated the expression of AKT2 in CRC cells; forced expression of miR-612 suppressed AKT2 protein levels, whereas inhibition of miR-612 promoted AKT2 expression, as well as the phosphorylation of AKT2 (Figure 3c). Furthermore, expression of miR-612 in human CRC samples was negatively related to AKT2 levels (Figure 3d). Collectively, these results indicate that AKT2 is a direct target of, and regulated by, miR-612.

### AKT2 reversed the impaired CRC cell growth mediated by miR-612

**Figure 4:**
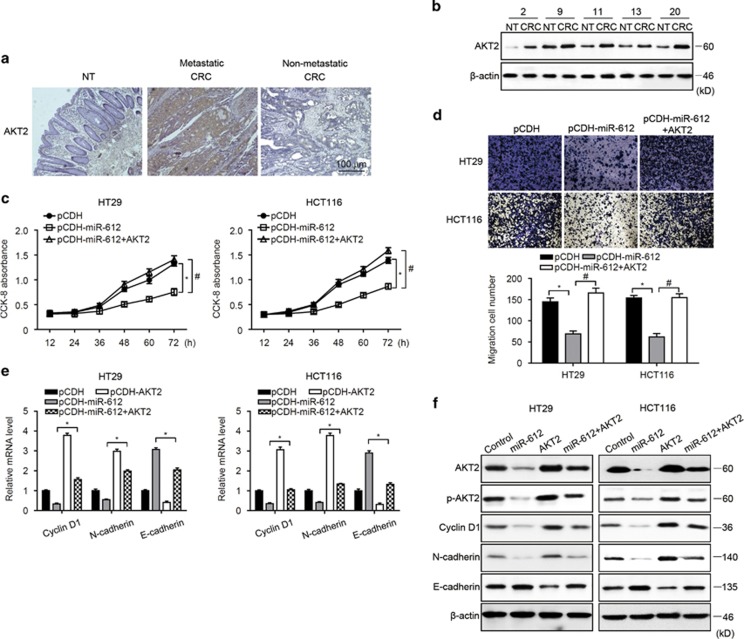
Forced expression of AKT2 rescued impaired CRC growth caused by miR-612. (**a**) AKT2 expression was detected in NT specimens, and metastatic and non-metastatic CRC specimens by immunohistochemistry. Scale bar, 100 *μ*m. (**b**) miR-612 expression was measured by western blotting in CRC and NT specimens from five random selected patients with metastatic CRC. (**c**) miR-612 and AKT2 were ectopically expressed in CRC cells. CCK-8 assay was conducted in the indicated cells (*n*=3). (**d**) miR-612 and AKT2 were ectopically expressed in CRC cells. The transwell assay was performed in the indicated cells. Representative images are shown on the top and summarized results are mean±S.D. from three independent experiments (*n*=3) on the bottom. (**e** and **f**) miR-612 and AKT2 were ectopically expressed in CRC cells. Cyclin D1, E-cadherin, and N-cadherin were detected by real-time PCR (**e**) and western blotting (**f**) in the indicated CRC cells (*n*=3). All the data were presented as mean±S.D. from three independent experiments. *^,#^*P*<0.05

We demonstrated above that miR-612 targeted and regulated the expression of AKT2. We therefore investigated the ability of AKT2 to alter the effect of miR-612 on CRC growth. First, we observed that AKT2 expression was markedly increased in metastatic CRC specimens compared with adjacent NT and non-metastatic CRC specimens by immunohistochemistry (Figure 4a) and western blotting (Figure 4b), demonstrating the opposite situation to miR-612. Then, we overexpressed AKT2 in two CRC cells by lentivirus infection, finding out that ectopic expression of AKT2 in miR-612-overexpressing CRC cells rescued cell growth (Figure 4c) and migration activity (Figure 4d). In addition, both mRNA and protein levels of cyclin D1 and N-cadherin were promoted by AKT2, although E-cadherin was inhibited (Figures 4e and f), which reversed the miRNA-612 effect. These results indicate that AKT2 induced proliferation and EMT progression in CRC cells and reversed the inhibitory effects of miR-612.

### miR-612 regulated CRC occurrence and metastasis *in vivo* by altering AKT2 expression

**Figure 5:**
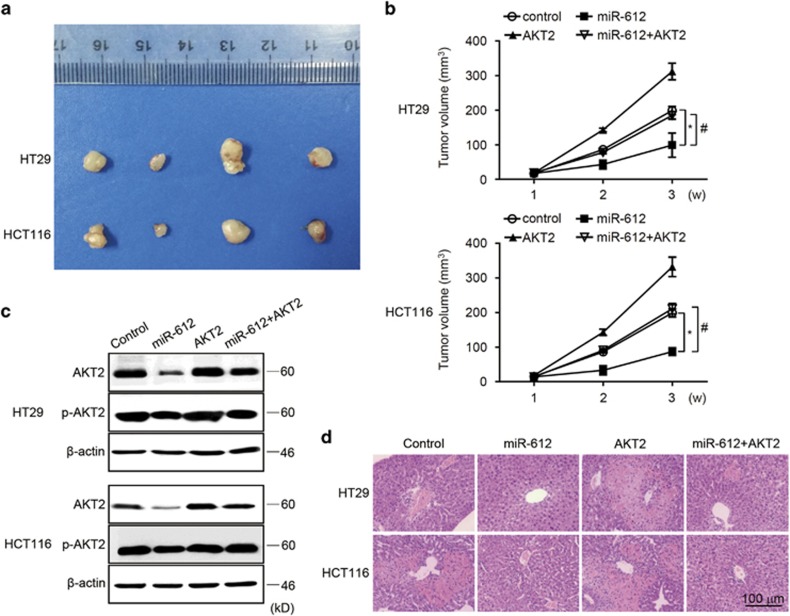
miR-612 suppressed tumor growth and metastasis through AKT2 *in vivo*. (**a**) CRC cells that stably overexpressed miR-612 or AKT2 were collected, suspended at the density of 1 × 10^6^, and inoculated subcutaneously into the right flank regions of 4-week-old male BALB/c nude mice. Xenograft tumors isolated from nude mice on day 14 in different groups. (**b**) Size of the tumors formed by subcutaneous injection was measured and calculated every 7 days, completing growth curve of xenograft tumors in different groups (*n*=6 mice per group). (**c**) The expression of AKT2 was detected by western blotting in the indicated groups of tumors. (**d**) CRC cells (2 × 10^6^) that stably expressed miR-612 or AKT2 were produced and injected into the spleen of BALB/c nude mice under anesthesia. Mice were killed 8 weeks after injection, and the livers were surgically excised and subjected to hematoxylin and eosin staining to detect the liver lesion caused by CRC metastasis. Scale bar, 100 *μ*m. All the data were presented as mean±S.D. from three independent experiments. ^*,#^*P*<0.05

We tested the AKT2-dependent effects of ectopic expression of miR-612 on tumor growth *in vivo*. CRC cells stably overexpressing miR-612, AKT2, or both miR-612 and AKT2, and control CRC cells were produced and injected subcutaneously into nude mice. Tumor sizes were measured every 7 days and the mice were killed and photographed at 21 days postimplantation. The tumors were significantly smaller in the miR-612 group compared with the control group, whereas the tumors in the AKT2 group showed the opposite trend (Figure 5a). The growth curve of different experimental groups also showed that tumors with high expression of miR-612 obtained the characteristic of slow growth and AKT2 rescued the impaired tumor growth (Figure 5b). Thus, forced expression of AKT2 totally blocked the inhibitory effect of miR-612 on tumor size. The tumors from different groups were subjected to western blotting, demonstrating that miR-612 significantly downregulated AKT2 protein levels, which effects were reversed by AKT2 overexpression (Figure 5c). These data demonstrate that miR-612 regulated CRC growth *in vivo* via AKT2. To clarify the impact of miR-612 on CRC metastasis, xenograft animal model of liver metastasis using the indicated CRC cells was therefore established, showing that the size of liver focal lesion because of colorectal tumor cell metastasis was significantly smaller by miR-612 overexpression, which was partially reversed by AKT2 overexpression (Figure 5d). These results collectively indicate that miR-612 can efficiently suppress tumor growth and liver metastasis *in vivo*.

## Discussion

Accumulating evidence supports miRNAs as effective molecular biomarkers for cancer diagnosis, prognosis, and therapy. Many miRNAs have been shown to be aberrantly expressed in CRC by miRNA-CHIP assays, and the functions of some miRNAs have already been clarified.^12^ Examination of the colorectal miRNAome revealed that miR-612 was downregulated in tumors versus non-tumor colorectal tissues, but its role in CRC occurrence remains unknown. Previous studies showed that miR-612 inhibited the capacity of tumorigenesis and metastasis of HCC by targeting AKT2.^21, 22^ Here we extended these findings to CRC occurrence and development, the underlying mechanism of which in not new. The current study demonstrated a critical role for miR-612 in CRC development. miR-612 expression was significantly lower in CRC tissues compared with NTs, and lower in metastatic compared with non-metastatic CRC. We also showed that miR-612 inhibited CRC cell proliferation and migration by suppressing the expression of AKT2. Importantly, AKT2 could totally reverse the effects of miR-612 on CRC suppression. These phenomena were validated in a mouse tumor xenograft model.

Surgery and multimodal treatments are the preferred strategies following an early-stage diagnosis of CRC, with a 5-year survival rate of ~100%, although the 5-year survival is significantly reduced for stage III CRC, which is characterized by lymph node metastasis. However, metastasis to distant organ sites (stage IV) is the most frequent cause of CRC-related deaths, with a 5-year survival rate of <5%. Approximately 50% of patients diagnosed with CRC die as a result of complications related to distant metastasis.^23, 24^ Metastasis is a multistep process in which malignant cells disseminate from the primary tumor to colonize distant organs, followed by proliferation, induction of angiogenesis and evasion of apoptotic death. CRC metastasis involves EMT, whereby tumor cells become more invasive and metastatic.^25, 26^ EMT is a biological process that drives polarized, immotile epithelial cells to undergo multiple biochemical changes to acquire a mesenchymal cell phenotype. The characteristic features of EMT are cell apolarity, loss of cellular adhesion, reduced expression of E-cadherin, and increased migratory capacity, as well as invasiveness. Abnormal activation of EMT contributes to some human pathologies such as tissue fibrosis, cancer cell invasion, and metastasis. In carcinomas, cancer cells can undergo EMT to escape the primary tumor, invade surrounding tissues, and eventually colonize remote sites via blood or lymphatic routes to generate metastases. Metastatic cells can then revert and reacquire epithelial characteristics, similar to cells in the primary tumor.^25, 26^ Several miRNAs have been shown to target families of EMT transcription factors. The miR-200 family, which includes miR-200a, miR-200b, miR-200c, miR-141, and miR-429, targets ZEB1 and ZEB2. Their expression is reduced during EMT, resulting in enhanced ZEB1 and ZEB2 levels and EMT progression.^16^ Snail is targeted by several miRNAs including miR-29b and miR-30a. Enhanced expression of miR-29b in metastatic prostate cancer cells accordingly reverses EMT and inhibits the invasive phenotype,^27^ whereas miR-30a expression is reduced during transforming growth factor-*β*-induced EMT in murine hepatocytes.^28^ According to our results, miR-612 suppressed CRC metastasis by inhibiting AKT2, which is also a contributor to EMT. However, further studies are needed to determine if other EMT-related proteins are regulated by miR-612.

AKT is an evolutionarily-conserved serine/threonine kinase involved in the PI3K/AKT signaling pathway, which regulates cellular processes such as cell proliferation, apoptosis, migration, and metabolism.^29, 30^ The PI3K/AKT signaling pathway is recognized as one of the most frequently activated signaling pathways in human cancers and forms a major link between oncogenic receptors and downstream prosurvival molecules. PI3K/AKT signaling modulates the expression of cell cycle- and cell apoptosis-related proteins such as cyclin D1, Bcl2, and Bax, as well as EMT-related proteins including N-cadherin and E-cadherin.^29, 30^ AKT comprises AKT1 (protein kinaseBα PKB*α*), AKT2 (PKB*β*), and AKT3 (PKB*γ*). AKT isoforms are aberrantly expressed in tumor conditions in tumor-specific manners. AKT1 amplification is commonly observed in gastric cancer cells, and knockdown of AKT1 increases the sensitivity of gastric cancer cells to cisplatin. AKT1 knockdown in gastric cancer cells also increases the expression of Bax and reduces the expression of Bcl2, thus increasing cell death *in vitro* and *in vivo*. In contrast, AKT2 is abnormally expressed in breast, ovarian and colon cancers, and AKT3 is amplified in breast and prostate cancers.^29, 30^ Although AKT1 and AKT3 are generally associated with other tumor types, they are highly conserved with AKT2 and involved in tumor proliferation and metastasis. Thus, it would be of interest to determine if they are also regulated by miR-612 in CRC development.

In summary, we demonstrated that miR-612 was downregulated in CRC tissues compared with NTs, and in metastatic compared with non-metastatic CRC. miR-612 impaired cell proliferation and migration mainly by inhibiting AKT2 *in vitro* and *in vivo*. The results of this study improve our understanding of the regulation of CRC development and provide potential new therapeutic targets for the management of CRC.

## Materials and Methods

### Clinical specimens and cell lines

Forty CRC and 40 adjacent NT specimens were collected from patients undergoing surgery with curative intent at Shanghai Jiao Tong University Affiliated Sixth People's Hospital, according to the Institutional Review Board-approved protocol, with 19 patients with metastatic CRC and 21 with non-metastatic CRC included in the study. All participants gave informed written consent before participating in this study. Histologic sections were reviewed by two expert pathologists to verify the histologic diagnosis. All tissues were immediately dissected, placed on ice, snap frozen in liquid nitrogen and stored at −80 °C until processing.

The normal HIEC line, the CRC cell lines HT29 and HCT116, and 293 T cells were all purchased from the American Type Culture Collection (Manassas, VA, USA) and were routinely cultured in RPMI-1640 medium (Invitrogen, Carlsbad, CA, USA) supplemented with 10% fetal bovine serum (FBS; Life Technologies, Carlsbad, CA, USA) in a humidified cell incubator with an atmosphere of 5% CO_2_ at 37 °C.

### Plasmid construction

The human miR-612 precursor and human AKT2 coding sequence were cloned into the lentivirus pCDH vector (System Biosciences, San Francisco, CA, USA) to generate stably transfected CRC cell lines. Virus was produced in 293 T cells co-transfected with lentiviral vector pCDH and packaging plasmid. Cells were incubated overnight at 37 °C and 5% CO_2_. The supernatant was collected 48 h posttransfection. The virus stock solution was then used to infect CRC cells. A fragment of the AKT2 3′-UTR containing either the predicted binding site for miR-612 or a mutated or deleted 3′-UTR was inserted into the psiCHECK2 vector for dual-luciferase reporter assay. Mutation and deletion of miR-612 binding sites in the AKT2 3′-UTR were achieved by site-directed mutagenesis using the QuikChangeH XL Site-Directed Mutagenesis Kit (Stratagene, Santa Clara, CA, USA) according to the manufacturer's instructions.

### Cell transfection

Oligonucleotides including miR-612 mimic and the miR-612 inhibitor anti-miR-612 were used (Thermo Scientific, Lafayette, CO, USA) for overexpression or inhibition of miR-612, respectively. For cell transfection assays, the synthetic oligonucleotides were transfected into cells using a Lipofectamine RNAiMAX Kit (Invitrogen) at about 50% confluence, according to the product manual. The media were changed 24 h posttransfection and the indicated cells were subjected to further investigations.

### RNA extraction and real-time PCR

Total RNA was extracted from cultured cells or CRC tissues using TRIzol (Invitrogen). Mature miRNA was detected by reverse transcription of miRNA using a TaqMan miRNA Reverse Transcription Kit (Applied Biosystems, Foster City, CA, USA). Real-time PCR was carried out using the appropriate TaqMan miRNA assay (Applied Biosystems) and a Prism 7500 instrument (Applied Biosystems). U6 was quantified as an endogenous control. mRNA expression was detected by reverse transcription of 500 ng of total RNA into cDNA using PrimeScript reverse transcriptase and random primers (TaKaRa, Otsu, Shiga, Japan), followed by real-time PCR with SYBR Green (Applied Biosystems). *β*-Actin was used as an endogenous control. Analyses were performed in triplicate and repeated at least three times. The primers for the genes of interest were as follows (5′–3′): E-cadherin, CTGCAGGTCTCATCATGGA and ACCTGTAGACCTCGGCACTG; N-cadherin, CCGTGAATGGGCAGATCACT and TAGGCGGGATTCCATTGTCA; cyclin D1, GCTGCGAAGTGGAAACCATC and CCTCCTTCTGCACACATTTGAA; and *β*-actin, CACCATGAAGATCAAGATCATTGC and GGCCGGACTCATCGTACTCCTGC.

### Western blotting

The cells were lysed with buffer containing 2% sodium dodecyl sulfate, 50 mM Tris-HCl (pH 6.8), 10 mM dithiothreitol, 10% glycerol, 0.002% bromophenol blue, and protease inhibitor mixture (Roche, Basel, Switzerland). Equal amounts of protein were separated by sodium dodecyl sulfate-polyacrylamide gel electrophoresis and transferred to a polyvinylidene difluoride membrane (Millipore, Billerica, MA, USA). After blocking in 5% nonfat milk or bovine serum albumin, the membrane was immunoblotted with antibodies and visualized with horseradish peroxidase-coupled secondary antibodies. The primary antibodies against AKT2, p-AKT2, cyclin D1, E-cadherin, N-cadherin, and *β*-actin were from Cell Signaling Technology (Danvers, MA, USA).

### Cell proliferation assays

Cell proliferation was determined using a CCK-8 (Dojindo Laboratories, Kumamoto, Japan). Briefly, CRC cells were seeded into 96-well plates at an initial density of 5000 cells per well. Cells transfected with anti-miR-612 were plated at day 1 after transfection. Ten microliters of the kit reagent were added to each well at 12, 24, 36, 48, 60, or 72 h after seeding, and all plates were scanned by a microplate reader (Thermo Scientific) after a further 2 h. Cell proliferation was evaluated by absorbance at 450 nm.

### Cell migration assays

Cell migration was analyzed using a Transwell Permeable Supports system (Corning Incorporated, Corning, NY, USA). Cells were seeded at a density of 1 × 10^4^ into the upper uncoated inserts with serum-free medium. Cells transfected with anti-miR-612 were plated at day 2 after transfection. Medium containing 10% FBS was added to the lower chamber. Cells were allowed to migrate for 12 h at 37 °C. Non-migrating cells on the upper surface of the membrane were gently removed. The remaining cells were fixed, stained, and analyzed by inverted microscopy. The migrating cells were counted in five randomly selected areas to evaluate the migration activity of the indicated cells.

### Luciferase reporter assays

Luciferase reporter assays were conducted using the Dual-Luciferase Reporter Assay System (psiCHECK2 vector; Promega, Madison, WI, USA). A fragment of the AKT2 3′-UTR containing either the predicted binding site for miR-612 or a mutated or deleted 3′-UTR was inserted into the psiCHECK2 vector. After verification by DNA sequencing, the psiCHECK2 vector containing either the wt, mutated, or deleted AKT2 3′-UTR was transfected into CRC cells with or without synthetic miR-612 mimic, using a Lipofectamine RNAiMAX Kit (Invitrogen) following the manufacturer's instructions. At 36 h after transfection, luciferase activity was detected using a dual-luciferase reporter assay system and normalized to *Renilla* activity. Data were normalized to the luciferase activity of cells transfection with miR control.

### Immunohistochemistry

CRC and NT specimens were fixed in 10% neutralized formalin and embedded in paraffin blocks. Sections can be sliced on microtome or cryostat and were then prepared for immunohistochemical examination. After deparaffinization and rehydration, antigen retrieval was performed by boiling samples in 10 mmol/l citrate buffer (pH 6.0) for 10 min. After inhibition of endogenous peroxidase activity for 30 min with methanol containing 0.3% H_2_O_2_, sections were blocked with 2% bovine serum albumin in phosphate-buffered saline for 30 min and incubated with AKT2 antibody (Cell Signaling Technology). The immune complex was visualized and nuclei were counterstained with hematoxylin.

### Tumorigenesis in nude mice

Male BALB/c nude mice (4 weeks of age) were purchased from the Shanghai Laboratory Animal Center of the Chinese Academy of Sciences (Shanghai, China). CRC cells (1 × 10^6^) stably expressing miR-612 or AKT2 were collected and inoculated subcutaneously into the right flank regions of mice. Tumor nodules were measured every 7 days using calipers. Mice were killed after 1 month and the tumor growth rate and rate of inhibition were calculated. Three independent experiments were performed for each experimental group.

### Animal model of liver metastasis

We determined the effect of miR-612 and AKT2 expression on CRC metastasis in xenograft mouse models of liver metastasis. CRC cells stably overexpressing miR-612, AKT2, or both miR-612 and AKT2, and control CRC cells, were produced by lentivirus infection. In the liver metastasis model, the indicated cells were suspended into single cell (2 × 10^6^) in 100 *μ*l phosphate-buffered saline and were slowly injected into the spleen of BALB/c nude mice under anesthesia. Mice were killed 8 weeks after injection, the livers were surgically excised and subjected to hematoxylin and eosin staining. The metastatic lesion was observed by microscope.

### Statistical analysis

All statistical analyses were performed using SPSS version 13.0 (SPSS Inc., Chicago, IL, USA). Values were presented as mean±S.D. from three independent experiments. Differences/correlations between groups were calculated using Student's *t*-tests or Spearman's correlation analysis. A *P-*value <0.05 was defined as significant.

## Figures and Tables

**Table 1:** Clinicopathologic characteristics of colorectal cancer patients

Clinical parameters	*N* (%)
*Sex*	
Male	25 (62.5%)
Female	15 (37.5%)
	
*Age (years)*	
≤60	14 (35.0%)
>60	26 (65.0%)
	
*Location*	
Ascending colon	3 (7.5%)
Transverse colon	5 (12.5%)
Descending colon	10 (25.0%)
Sigmoid colon	19 (47.5%)
Rectum	3 (7.5%)
	
*TNM stage*	
I	5 (12.5%)
II	14 (35.0%)
III	11 (27.5%)
IV	10 (25.0%)
	
*Histologic differentiation*	
Well	9 (22.5%)
Moderately	17 (42.5%)
Poorly	14 (35.0%)
	
*Lymphatic metastasis*	
Yes	19 (47.5%)
No	21 (52.5%)
	
*Distant metastasis*	
Yes	16 (40.0%)
No	24 (60.0%)

Abbreviation: TNM, tumor lymph node metastasis

## Abbreviations

anti-miR-612: miR-612-specific antisense oligonucleotide inhibitor
CCK-8: Cell Counting Kit-8
CRC: colorectal cancer
EMT: epithelial–mesenchymal transition
FBS: fetal bovine serum
HCC: hepatocellular carcinoma
HIEC: human intestinal epithelial cell
miRNA: microRNA
NT: normal tissue
PI3K: phosphoinositide 3-kinase
S.D.: standard deviation
TNM: tumor lymph node metastasis
UTR: untranslated region
wt: wild type
